# Prognostic meta-signature of breast cancer developed by two-stage mixture modeling of microarray data

**DOI:** 10.1186/1471-2164-5-94

**Published:** 2004-12-14

**Authors:** Ronglai Shen, Debashis Ghosh, Arul M Chinnaiyan

**Affiliations:** 1 Department of Biostatistics, University of Michigan, Ann Arbor, MI 48109, USA; 2 Department of Pathology, University of Michigan, Ann Arbor, MI 48109, USA; 3 Department of Urology, University of Michigan, Ann Arbor, MI 48109, USA; 4 The Comprehensive Cancer Center, University of Michigan, Ann Arbor, MI 48109, USA

## Abstract

**Background:**

An increasing number of studies have profiled tumor specimens using distinct microarray platforms and analysis techniques. With the accumulating amount of microarray data, one of the most intriguing yet challenging tasks is to develop robust statistical models to integrate the findings.

**Results:**

By applying a two-stage Bayesian mixture modeling strategy, we were able to assimilate and analyze four independent microarray studies to derive an inter-study validated "meta-signature" associated with breast cancer prognosis. Combining multiple studies (*n *= 305 samples) on a common probability scale, we developed a 90-gene meta-signature, which strongly associated with survival in breast cancer patients. Given the set of independent studies using different microarray platforms which included spotted cDNAs, Affymetrix GeneChip, and inkjet oligonucleotides, the individually identified classifiers yielded gene sets predictive of survival in each study cohort. The study-specific gene signatures, however, had minimal overlap with each other, and performed poorly in pairwise cross-validation. The meta-signature, on the other hand, accommodated such heterogeneity and achieved comparable or better prognostic performance when compared with the individual signatures. Further by comparing to a global standardization method, the mixture model based data transformation demonstrated superior properties for data integration and provided solid basis for building classifiers at the second stage. Functional annotation revealed that genes involved in cell cycle and signal transduction activities were over-represented in the meta-signature.

**Conclusion:**

The mixture modeling approach unifies disparate gene expression data on a common probability scale allowing for robust, inter-study validated prognostic signatures to be obtained. With the emerging utility of microarrays for cancer prognosis, it will be important to establish paradigms to meta-analyze disparate gene expression data for prognostic signatures of potential clinical use.

## Introduction

DNA microarray analysis has been shown to be a powerful tool in various aspects of cancer research [1]. With the increasing availability of published microarray data sets, there is a tremendous need to develop approaches for validating and integrating results across multiple studies. A major concern in the meta-analysis of DNA microarrays is the lack of a single standard experimental platform for data generation. Expression profiling data based on different technologies can vary significantly in measurement scale and variation structure. It poses a great challenge to compare and integrate results across independent microarray studies. In a recent study of diffuse large B cell lymphoma (DLBCL), Wright et al. [2] sought to bridge two different microarray platforms by validating findings from a cDNA lymphochip microarray using an independent dataset generated using Affymetrix oligonucleotide arrays. Although the idea of training and testing classifiers is frequently used for discriminant analysis, this application to distinct expression array platforms is less common.

More systematic approaches have been proposed for integration of findings from multiple studies using different array technologies. Rhodes et al. [3] have proposed methods to summarize significance levels of a gene in discriminating cancer versus normal samples across multiple gene profiling studies. By ranking the q-values [4] from sets of combinations, a cohort of genes from the four studies was identified to be abnormally expressed in prostate cancer. Choi et al. [5] suggested combining effect size using a hierarchical model, where the estimated effect size in individual studies follows a normal distribution with mean zero and between study variance *τ*^2^. The effect size was defined to be the difference between the tumor and normal sample means divided by pooled standard deviation. From a Bayesian perspective, Wang et al. [6] used data from one study to generate a prior distribution of the differences in logarithm of gene expression between diseased and normal groups, and subsequent microarray studies updated the parameter values of the prior. Assuming a normal error distribution, the differences were then combined to form a posterior mean. Although phrased using different model frameworks, these methods are similar in the spirit of combining the standardized differences between two sample means across multiple studies. It has been shown, however, that the overlap between significant gene detection on different array platforms is only moderate due to low comparability of independent data sets [7]. The large variability brought in by microarray datasets using different platforms is expected to affect the sensitivity and specificity of summary statistics constructed in various ways across studies. Given the inherent differences of the microarray techniques, heterogeneity of the sample populations, and low comparability of the independently generated data sets, meta-analysis of microarrays remains a difficult task.

A recent study proposed a Bayesian mixture model based transformation of DNA mi-croarray data with potential features applicable to meta-analysis of microarray studies [8]. The basic idea is to estimate the probability of over-, under- or baseline expression for gene sample combinations given the observed expression measurements. With data-driven estimation of these quantities, one can translate the raw expression measurement into a probability of differential expression. As a result, *poe *(i.e., probability of expression) was introduced as a new scale and used in the context of molecular classification [8]. The platform-free property of this scale, however, motivated us to incorporate *poe *in a framework to meta-analyze microarray data. Several desirable features of using *poe *as a new expression scale include the following: 1. *poe *provides a scaleless measure and thereby facilitates data integration across microarray platforms; 2. *poe *is a model-based transformation with direct biological implications in the context of gene expression data, as it is estimated based on a method that adopts an underlying mixture distribution that accommodates over-, under-, and unchanged expression categories; 3. *poe *unmasks differential expression patterns in microarray data by offsetting the influence of extreme expression values [9]; 4. Data integration based on *poe *allows merging of samples on the unified scale rather than using gene-specific summaries.

In recent publications of breast cancer microarray studies, several groups have explored the hypothesis that the capacity to metastasize is intrinsic to the tumor and therefore can be revealed by gene expression pattern. Four independent studies have correlated gene expression profiles generated from distinct DNA microarray platforms to breast cancer prognosis [10-13]. Among the four, Sorlie et al. [10] and Sotiriou et al. [12], both cDNA microarray studies, applied unsupervised clustering and identified several breast cancer subtypes characterized by differential expression of a cohort of genes. Further, they correlated the tumor subtypes derived from the expression profile with survival outcome and in both cases found that, as expected, the ERBB2+ subtype correlated with shorter survival times. On the other hand, van't Veer et al. [11], an inkjet oligonucleotide array study, and Huang et al. [13], an Affymetrix GeneChip study, have built classification models based on gene expression profiles to predict 5-year or 3-year recurrence status. In all four studies, however, the authors explored a common hypothesis that molecular profiles were able to provide a more accurate prediction of patient survival compared with clinical/pathological parameters. These studies therefore provided an excellent basis for developing a meta-analysis of microarrays with regard to disease prognosis.

In this proof-of-concept study, we propose a two-stage meta-analysis of microarrays based on *poe*. We applied our method to the aforementioned breast cancer DNA microarray data sets. With the strength of the *poe *transformation and data integration, our goal was to develop an inter-study validated meta-signature that predicts relapse-free survival in breast cancer patients with improved statistical power and reliability.

## Results

### Development of the two-stage Bayesian mixture modeling approach for the meta-analysis of microarray data

**Figure 1:**
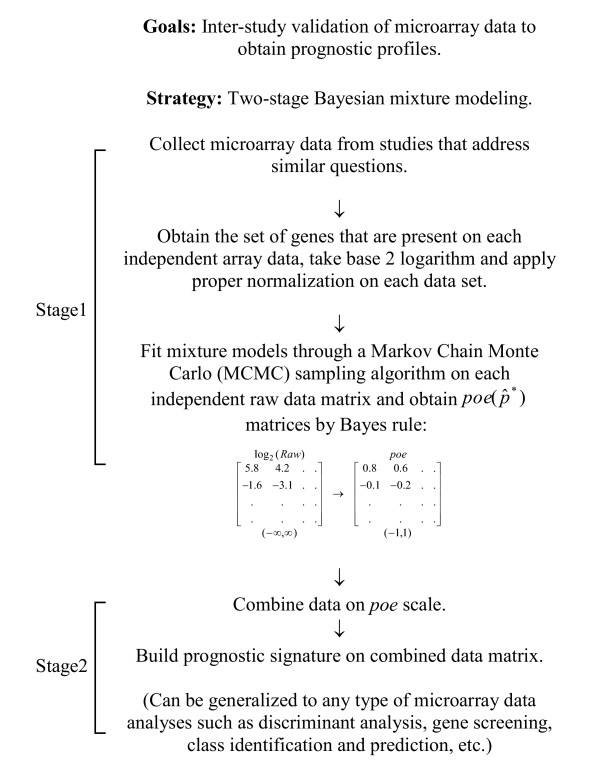
Meta-analysis of microarray data using a two-stage mixture model approach.

Figure 1 outlines the two-stage Bayesian mixture modeling strategy. The idea is to build a scale that can be combined across different microarray platforms, and therefore allows simultaneous examination of independent data sets. The stage 1 of the analysis involves data-driven estimation of posterior probability of differential expression, namely *poe*. The Bayesian hierarchical model employed for estimation borrows strength across genes by assuming further distributions for the gene-specific parameters (see Methods). For data integration purposes, we focused on a common set of 2,555 genes that were profiled in each of the four studies. Although the cost for compiling common genes is a loss of potential predictive features, it is not unreasonable to assume, given the analogous hypothesis explored in each study, that the common set represents the most relevant genes of interest for breast cancer prognosis. The resulting values of *poe *represent signed probability of differential expression for gene *j *in sample *i*, and thus provide a unified measure across studies. Further, the transformation improves contrast in each data set by removing the influence of extreme expression values. In stage 2, the expression profile of tumor samples from multiple studies were combined on the *poe *scale to generate a meta-cohort. The benefit of data integration using *poe *is twofold. First, it improves power of statistical analysis by increasing the sample size. Such integration of independent data sets renders sensitivity to those small yet consistent expression changes for certain genes. Second, it reduces the chance of false positive features due to artifacts from a single study, and allows reliable findings across studies. In this paper, we integrated four breast cancer microarray data sets of distinct platforms (Table 1), and developed a prognostic meta-signature for disease recurrence.

**Table 1:** Breast cancer gene expression data sets used in the prognostic meta-analysis. Bad outcome (Y = 1) is defined as recurrence during follow-up, and good outcome (Y = 0) is defined as remaining recurrence-free for at least three years.

Authors	Array platform	Number of array elements	Sample size (n)	Good outcome (n_0_)	Bad outcome (n_1_)
Sorlie *et al.*	Spotted cDNA	8102	58	23	35
van't Veer *et al.*	Inkjet oligonucleotide	25000	78	44	34
Sotiriou *et al.*	Spotted cDNA	7650	98	53	45
Huang *et al.*	Affymetrix chip	12625	71	36	35

### Building a gene expression meta-signature for breast cancer prognosis

In the second stage of the analysis, We assessed the performance of the genes found using the meta-analysis methods based on classification accuracy. A complication is that while most methods of classification deal with data from two populations, the response with which we wish to build classifiers to predict is time to breast cancer recurrence. While the ideal data would have information on time to recurrence on all subjects (potentially censored), not all studies have the time to recurrence information available and instead provide data on recurrence within a certain time interval (e.g., recurrence within five years versus no recurrence within five years). To deal with this issue, we utilized a dichotomization where a bad outcome is recurrence during followup and a good outcome is remaining recurrence-free for at least three years. The additional constraint for the good outcome group is to reduce potential bias introduced by short censoring due to insufficient length of follow-up. This is particularly relevant in cross-study analysis, given the heterogeneity in patient recruitment criteria and study designs. Accordingly, of the combined meta-cohort (n = 305) of breast cancer patients, 48.9% were in the poor outcome group, whereas 51.1% in the good outcome group. The sample sizes for each study are shown in Table 1.

Each gene was then associated with the recurrence status by a logistic regression within a leave-one-out cross validation scheme, and rank-ordered by the significance level of the coefficient. As a result, 23 genes held up as significant predictor of recurrence (*P *≤ 0.001) in all cross-validation steps, representing a cohort of essential genes strongly associated with breast cancer recurrence. By random chance, there would be on average 2.5 genes to be found significant at *P *≤ 0.001 in a set of 2,555. By finding 23 genes with a *P *≤ 0.001, it is clear that there are much more predictive features than would be expected by random chance.

To identify a prognostic meta-signature, we define a risk index (RI) as a linear combination of the *poe *profile and the coefficient estimates from the univariate logistic regression for each gene *j*. Large positive values of *RI *indicate high risk of failure, whereas large negative values of *RI *indicate low risk of failure. Classification of sample *i *to the risk groups is then based on the *i*^*th *^leave-one-out risk index. The classifier is  = *I*{*RI*_*i *_>*c*}, with *c *being the empirical quantiles of the risk indices. The number of genes in a classifier is treated as a parameter and optimized to minimize the prediction error rates. More details on building a classifier at the second stage are described in the Methods section.

### The 90-gene expression meta-signature predicts clinical outcome in breast cancer patients

By minimizing the misclassification error, we obtained a 90 gene meta-signature that reliably predicts outcome in the meta-cohort. This meta-signature classified 122 patients into a high risk group, where 84 (69%) of them had a recurrence. On the other hand, the signature classified 183 patients into a low risk group, where 118 (64%) of them did not recur by the end of the followup. By cross-tabulating the risk groups predicted by the meta-signature and the actual recurrence status, we obtained an estimated odds ratio of 4.0 (95% CI: 2.5–6.5, *P *< 0.0001). In spite of the heterogeneity of the combined patient population, the meta-signature predicted the odds of recurrence for a patient showing a high risk signature as four times of the odds of recurrence for a patient showing a low risk signature. Several studies have implicated that the lymph node status is one of the principal clinical factors to classify patients in relation to the risk of relapse of breast cancer [14-16]. Although there have been controversial findings with regard to its predictive values in breast cancer survival outcome, we have shown in the meta-cohort that the nodal status is a significant risk factor of recurrence. The estimated odds of recurrence for node-positive patients is two times higher than the odds of recurrence for node-negative patients (95% CI:1.3–3.2, P = 0.002) in the combined samples.

**Figure 2:**
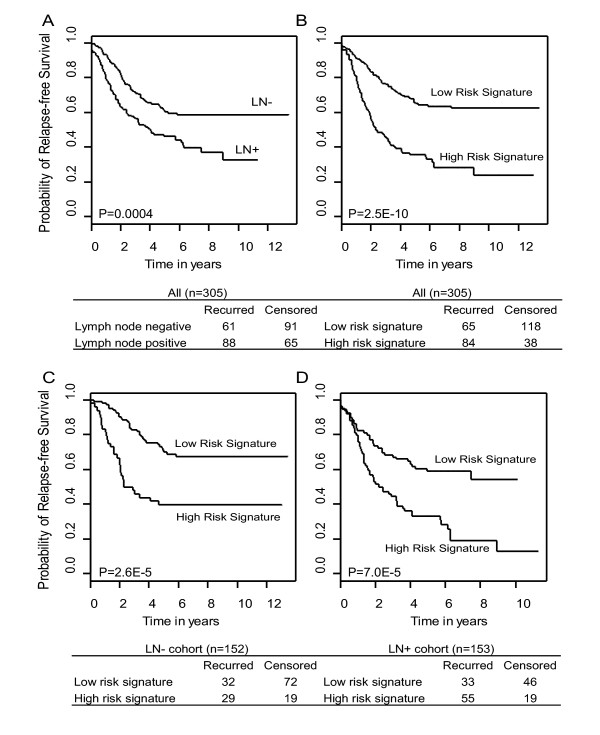
The 90-gene meta-signature displayed greater performance than nodal status in predicting relapse-free survival in breast cancer, and it further predicts survival outcome in nodal status sub-cohorts. (A) Lymph node status correlates with survival outcome (*P *= 0.0004). (B) The meta-signature correlates with survival outcome (*P *= 2 × 10^-10^). (C) The meta-signature differentiates risk groups in nodal negative patients (*P *= 2.6 × 10^-5^). (D) The meta-signature predicts risk groups in nodal positive patients (*P *= 7.0 × 10^-5^).

Kaplan-Meier analysis provides further evidence that the meta-signature was a significant prognostic index of breast cancer recurrence in the meta-cohort (Figure 2). The estimated three-year survival rate was 76.0%(± 3.2%) for low risk signature and 45.9%(± 4.5%) for high risk signature. Nodal status, on the other hand, was less discriminative at the three-year time point with an estimated survival rate of 71.7%(± 3.7%) for lymph node negative patients and 56.2%(± 4.0) for lymph node positive patients. Node-negative patients, although generally considered to be at low risk of recurrence, are heterogeneous in disease progression. About one third of node-negative patients develop local recurrence [17]. Many studies have therefore explored the potential of using molecular biomarkers to further differentiate patient survival outcome in nodal negative cohort [18-21]. As shown in Figure 2C and 2D, the meta-signature further differentiated 48 (31.6%) of the LN- patients to be at higher risk of recurrence during followup (*P *< 0.0001). Similarly for nodal positive patients, a cohort thought to be at high risk of recurrence, the meta-signature identified 79 (51.6%) of the LN+ patients to have, in fact, lower recurrence risk over time (*P *< 0.0001, Figure 2D). In contrast, nodal status failed to maintain its predictive power after controlling for the meta-signature risk groups (*P *= 0.05 and 0.12 in low risk signature and high risk signature group respectively). A multivariate logistic regression model suggested that the meta-signature is an independent predictor of the recurrent status with respect to nodal status in the meta-cohort (OR = 3.7(2.3–6.1), *P *< 0.0001).

### Comparison of the meta-signature to the study-specific signatures

To comprehend the potential gains of such two-stage meta analysis over individual analysis in each single study cohort, we constructed study-wise gene expression signatures using the same method. By minimizing the misclassification errors, we obtained a signature consisting 10, 60, 100, and 130 genes for Sorlie, van't Veer, Sotiriou, and Huang study cohort respectively (Additional file 5). The results of the classifiers are summarized in Table 2. In fact, not only did the size of the study-specific signatures vary significantly, but the elements of the signatures had very little overlap. At most two genes appeared in more than one signature among the four. In addition, signature identified in one study tended to have poor performance in other studies. Table 3 lists the estimated odds ratios for disease outcome and risk groups predicted by a gene expression signature. An individual signature identified in one study cohort demonstrated considerable shrinkage in the odds ratio estimates and non-significant 95% confidence intervals in the validation studies, indicating significantly reduced discriminative power in the testing cohorts. Kaplan-Meier analysis provided further evidence that the study-specific signatures performed poorly in pairwise cross-validations (Additional file 6).

**Table 2:** Comparisons of the number of genes (Size), the number of elements overlap with the meta-signature (overlap), and the prediction error rates for the signatures identified in individual study cohort and in the meta-cohort.

	**Sorlie**	**van't Veer**	**Sotiriou**	**Huang**	**Meta-cohort**
Size	10	60	90	140	90
Overlap	4	14	19	6	--
Prediction error rate	0.28	0.29	0.35	0.18	0.33

**Table 3:** Comparison of the performances of the individual signatures and the meta-signature in each single study cohort. Table lists odds ratios (95% confidence interval) comparing the odds of actual recurrence for those being classified as high risk to the odds of recurrence for those being classified as low risk of recurrence by each signature.

	**Cohort**			
**Signature**	Sorlie (n = 58)	van't Veer (n = 78)	Sotiriou (n = 98)	Huang (n = 71)
Sorlie (D = 10)	**18.6 (5.0, 69.5)**	2.1 (0.8, 5.4)	2.3 (1.0, 5.3)	10.87 (3.5, 33.8)
van't Veer (D = 60)	3.1 (1.1, 9.2)	**10.6 (3.3, 33.9)**	4.1 (1.7, 9.7)	1.3 (0.5, 3.4)
Sotiriou (D = 100)	1.7 (0.6, 5.0)	3.5 (1.4, 8.9)	**7.8 (3.0, 20.1)**	1.5 (0.6, 3.7)
Huang (D = 130)	5.1 (1.6, 15.7)	2.3 (0.9, 5.6)	0.9 (0.4, 2.0)	**184.9 (30.1, 1137.2)**
Meta (D = 90)	**25.0 (4.2, 149.0)**	**4.1 (1.6, 10.6)**	**6.0 (2.5, 14.5)**	**5.8 (2.1, 16.5)**

D is the number of genes in a signature. n is the sample size for each cohort.

**Figure 3:**
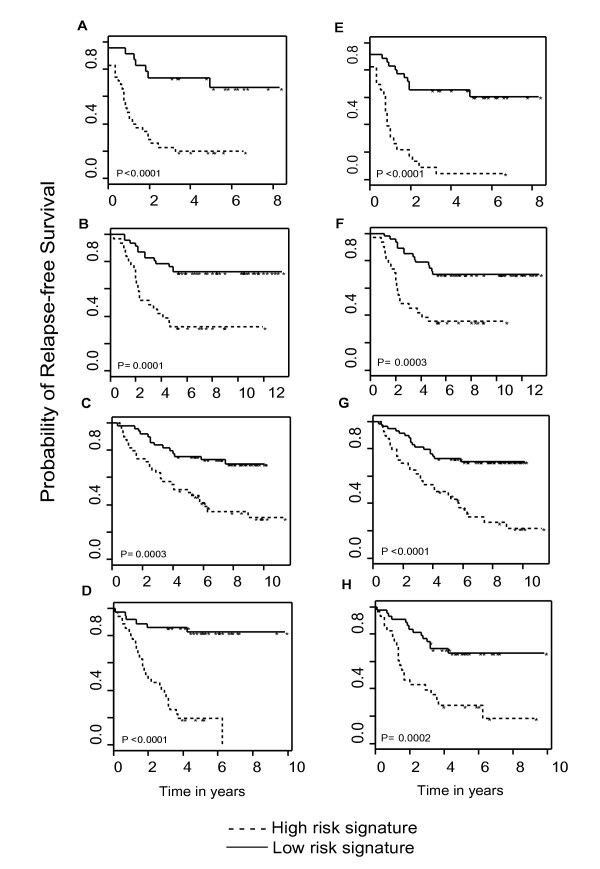
The 90-gene meta-signature achieves similar or better performance than the individually optimized signatures. A and E compare the Kaplan-Meier curves stratified by high versus low risk group predicted by the study-specific signature and by the meta-signature respectively in the Sorlie study cohort; B and F show similar comparison in the van't Veer study cohort; C and G show similar comparison in the Sotiriou study cohort; and D and H show comparison in the Huang study cohort.

Meta-analysis accounts for such heterogeneity of the individual signatures in two ways. First its overlap with the study-specific signatures ranged from 3–40%. The excluded genes are likely to be cohort-specific findings that can not be replicated. Second, the meta-signature recruited 41 genes not previously picked by any of the single cohort signature, likely representing predictive features with small but consistent effects previously masked in single studies. When examining the performances of the gene signatures, the meta-signature showed a comparable or better performance compared with the individually optimized signatures both in the odds ratio estimates (Bottom row of Table 3) and in Kaplan-Meier analysis (Figure 3). This shows that the meta-signature can serve as a common breast cancer recurrence index that is able to predict patient survival in heterogeneous sample populations. When a gene signature built in one study cohort performs differently in another, such meta analysis provides a solution to identify a cross-study validated expression signature that holds across independent samples.

### Comparison of data integration based on poe transformation and simple linear rescaling

An alternative approach to integrating data across multiple datasets is to perform a study-wise global normalization. For one study, let  be the globally scaled expression value for gene *j *in sample *i*. Each study dataset is then standardized to have zero mean and unit standard deviation. The linearly rescaled values can also be used for data integration purposes in that expression values generated from different array platforms are standardized to a common scale.

Such an approach is less computationally challenging compared to the mixture model-based rescaling described in the previous sections. However, there are several advantages to the mixture model-based transformation. First, the method incorporates biological information into estimating the posterior probabilities of expression. The transformed values carry meaningful interpretations as signed probabilities of differential expression of a gene in a particular sample. Second, the underlying normal and uniform mixture distributions give equal density in the tails and is effective in reducing the influence of extreme expression values. And third, the Bayesian hierarchical modeling approach borrows strength across genes resulting in shrinkage-type estimators for a large correlated gene-specific parameter vector. This is a method in which the high dimensional gene expression data are denoised.

**Figure 4:**
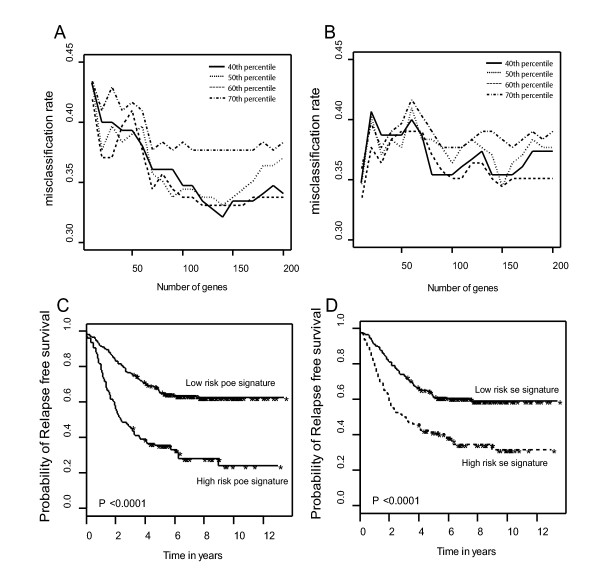
Comparison of model performances based on data integrated by *poe *transformation (A and C) and global standardization (B and D). A set of top 10 to 200 genes were used in a classifier to construct risk index and 40^*th *^to 70^*th *^percentiles of the cross-validated RIs were then used to dichotomize samples into a high risk or a low risk group. A. Misclassification rates based on *poe *transformation and B. based on global standardization. C. Performance of the 90-gene signature built on *poe *and D. built on global standardized data in differentiating patients at low risk of recurrence from those at high risk of recurrence.

To study the benefit of data integration based on *poe *compared to that based on the linearly rescaled values, we compared the model performances based on data integration by these two methods. Figure 4A shows that with the *poe *transformation, misclassification rates steadily decreases as more genes are used in the classifier. Performance based on linearly rescaled data (Figure 4B), however, is unpredictable. Figure 4C and 4D uses a 90-gene meta-signature based on *poe *and based on the global standardization respectively in predicting survival. The signature based on *poe *is noticeably better than the signature based on global standardization in differentiating patients at low risk of recurrence from those at high risk of recurrence. Taken together, the *poe *transformation outperforms the linear rescaling method in combining multiple microarray data sets. The meta-signature identified based on *poe *values therefore offers more reliable prediction of recurrence-free survival in the meta-cohort.

### The meta-signature displays two distinct expression patterns

**Figure 5:**
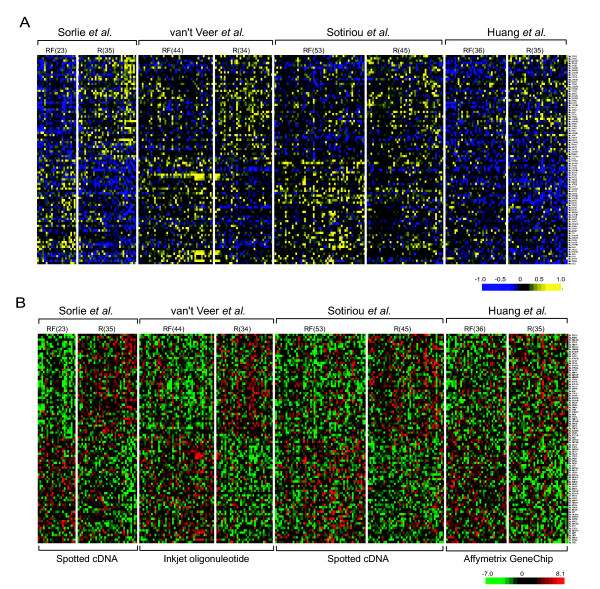
The 90 gene meta-signature displayed two distinct patterns of expression in breast cancer groups. (A) Heat map representation of differential expression probabilities for the 90 gene meta-signature across the combined samples. The top set of genes showed consistently high probability of over-expression (yellow) in the poor outcome group, and the bottom set of genes showed consistently high probability of down-regulation (blue) in the poor outcome group. (B) Heat map of log-transformed raw data. Individually generated heat maps of the raw measurements of gene expression confirmed the distinct expression patterns of the meta-signature from independent studies. Red represents up-regulation while green represents down-regulation. R (recurred) – poor outcome group; RF (recurrence-free) – good outcome group.

A heat map representation of the *poe *profile for the 90 gene meta-signature revealed two distinct patterns of differential expression (Figure 5A). Genes in the top half of the matrix displayed consistently high probability of over-expression (yellow) in the recurrent samples (R). On the other hand, genes in the bottom half displayed great probability of under-expression (blue) in the recurrent group. Individually generated heat maps of the raw data confirmed such distinct patterns at raw measurement levels (Figure 5B). Functional annotation revealed genes involved in many important biological processes such as cell cycle regulation (e.g., CDC28 protein kinase regulator subunit 2), cell adhesion (e.g., chemokine C-X3-C motif receptor 1), and apoptosis (e.g., secreted frizzled-related protein 4). A complete list of the meta-signature genes can be found in the Additional file 7. Some of the genes in the meta-signature were previously shown to correlated with breast cancer survival outcome. For example, Keyomarsi et al. [22] demonstrated the association of the cell cycle regulator cyclin E and death due to breast cancer.

### Enriched functional classes in the meta-signature

**Figure 6:**
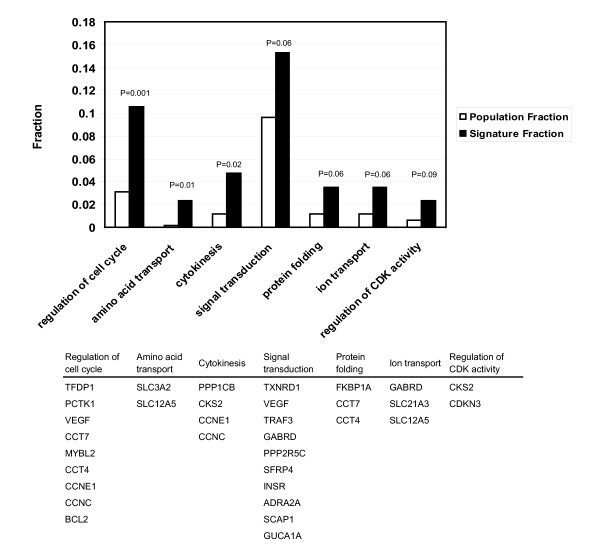
Top seven over-represented functional classes in the meta-signature. Black bars represent proportion of genes associated with each of the GO terms among the meta-signature, and white bars represent the corresponding proportion among the total study population of 2555 genes. P-value represents the significance of over-representation based on a hypergeometric distribution, and is calculated as the probability of observing larger proportion of a particular functional group genes in the meta-signature than in the entire gene set. The meta-signature genes are listed under each functional class.

To gain a better understanding of the processes related to disease recurrence, we examined whether a particular functionally defined biological process is enriched in the recurrence signature. Each of the ninety genes were mapped to Gene ontology (GO) terms and then grouped by functional classes. Based on the hypergeometric distribution, we calculated the significance of over-representation of a particular process in the signature. Figure 6 demonstrated the top seven enriched functional groups in the meta-signature, comparing the total proportion (out of 2310 annotated) and the signature proportion (out of 85 annotated) of genes in each group. Cell cycle regulation is the most highly over-represented category (P = 0.001). All genes under this category except BCL2 displayed increased expression level, reflecting elevated cell cycle activities. Signal transduction represents the largest functional class over-represented in the meta-signature. Genes involved in signalling pathways that regulate cell growth (VEGF, PPP2R5C), immune response (TRAF3), apoptosis (SFRP4), and other processes are found to constitute 15.7% of the meta-signature compared to the 9.7% in the entire gene set (the common set).

## Discussion

Several important issues to consider when integrating microarray studies include use of different gene expression measurement scales, varying analytical power and reliability of the results for individual studies. To account for these issues, we proposed a two-stage mixture modeling strategy, the strength of which was built on the mixture model based transformation and the subsequent data integration on the *poe *scale. In particular, *poe *provides a unified platform-free scale, and simultaneously enhances the intrinsic contrast in the expression data. Furthermore, combining sample pools on the *poe *scale mitigates the influence of potential artifacts from a single study. The benefit of such data integration is reflected on two counts. One, integrated sample cohorts improve the reliability of the findings by guarding against false positive results from a single study. Two, it increases the statistical power to detect small consistent effects that can be otherwise masked by inadequacy of the sample size of an individual data set. By implementing this modeling approach, we were able to combine information from four microarray studies to build an inter-study validated meta-signature for predicting survival in breast cancer patients.

As described earlier, a common set of 2555 genes was used in this meta-analysis, as it is important to provide the same context for data-driven estimation of the posterior probabilities. Although we assume the common set comprises the most biologically relevant genes, the loss of potential predictive genes, however, may offset the statistical power of the analysis. For example, one of our recent studies has established the polycomb protein EZH2 to be an independent predictor of breast cancer survival outcome[23]. This gene was filtered out of the meta-analysis as one of the studies [12] did not profile EZH2. However, in each of the other three studies where EZH2 was profiled on the array, its expression level was found to correlate with survival (data not shown), which confirmed its role as a prognostic marker. Alternative approaches to allow genes profiled in some studies but not others is a topic for future research.

Functional annotation of the meta-signature revealed genes such as Cyclin E and BCL2, which were previously shown to be correlated with survival outcome in breast cancer [22,24]. A strength of the inter-study validated signature is the capability of recruiting genes which may not be significant in one study due to limiting sample size or artifacts of the experiments. In this sense, the meta-signature will be more stable and less subjective to variations in subsets of the samples. As a result, the predictive genes in a meta-signature may carry more reliable information about tumor progression and patient survival.

In conclusion, a distinction of the analysis presented here relative to those by other authors [3,6] is that we sought to find genes that were predictive of recurrence rather than predictive of diseased versus nondiseased status. Given the heterogeneity of the tumors with respect to treatment response and survival outcome, a prognostic prediction analysis is generally more difficult because it is a more complicated phenotype. Further, a prognostic signature (classifier) of failure risk trained in one cohort is often times difficult to validate in independent cohorts. The meta-analysis method presented here may potentially provide more powerful gene signatures that are predictive of prognosis because they are validated across multiple studies.

## Methods

### Data collection and preparation

The breast cancer microarray data sets were obtained at the author's websites from four recently published studies [10-13]. Each data were preprocessed, either by a lowess normalization for two-channel microarray data [25] or a robust analysis for Affymetrix data [26]. We filtered for a common set of 2,555 genes from these four studies by Unigene Cluster IDs. Each data matrix of the 2,555 genes was then normalized by median centering and dividing by the standard deviation for each gene. Missing data were imputed by the k-nearest neighbors imputation algorithm [27].

### Mixture modeling of microarray data

Each of the four raw data sets was treated as an expression matrix *X *with elements *x*_*ij*_, where *i *= 1, ..., *m*_*k*_, *j *= 1, ..., *n *(*k *= 1, .., 4 and *n *= 2,555). The expression measurement *x*_*ij *_can be the ratio of the two fluorescent dye hybridization intensities for the spotted cDNA arrays[10,12] and the Intjek oligonucleotide array [11], or averaged difference between the perfect match and mismatch probe hybridizations for the Affymetrix gene chip [13]. Let *E *be a latent class variable, and *e*_*ij *_indicates over-, under- or normal expression for each entry of the R matrices. We have:



The values of *e*_*ij *_are latent and not directly observed from the data. We were interested in estimating the probabilities of *e*_*ij *_being 1 or -1 given the observed raw expression *x*_*ij*_, which were denoted as  and . Estimates of these latent quantities were obtained under a Bayesian mixture model setting. In particular, we assume the raw expression *x*_*ij *_falls into one of the three expression categories. For each gene *j*, the expression then arises from a mixture of three distributions:

(*x*_*ij*_|*e*_*ij *_= 1) ~ *f*_1,*j*_(·), (*x*_*ij*_|*e*_*ij *_= 0) ~ *f*_0,*j*_(·), and (*x*_*ij*_|*e*_*ij *_= -1) ~ *f*_-1,*j*_(·).

In the mixture, *f*_1,*j*_, *f*_0,*j *_and *f*_-1,*j *_are the density functions of the following distributions:



respectively. Here, U refers to a uniform distribution and N refers to a normal distribution. *α*_*i *_+ *μ*_*j *_is both the mean of the normal distribution and the threshold point in the uniform distribution. *μ*_*j *_is the gene effect and *α*_*i *_is the sample effect. The  and  provide limits to the uniform distribution in the mixture, and are set to be at least 3*σ*_*j*_.  = *P*(*e*_*ij *_= 1) and  = *Pe*_*ij *_= -1) are the multinomial probabilities for *e*_*ij*_. With the specifications of models, we can calculate the latent quantities by Bayes' rule:





By noting that the supports for the two uniform distributions are disjoint, the probabilities of differential expression are mutually exclusive with the forms:



A one dimension measure can thus be constructed as *poe *= *p*^+ ^- *p*^-^. As a result, *poe *ranges from -1 to 1, and can be interpreted as the signed conditional probability of differential expression.

To borrow strength across genes, the estimation of the gene-specific parameters was formulated under a Bayesian hierarchical model setting. Given the large amount of parameters, prior distributions were specified to model the variation of the gene-specific parameter estimates, in particular,



We followed the recommendations of Parmigiani et al. [8] in terms of the prior choices. A Metropolis-Hastings MCMC sampling algorithm was then implemented to approximate the posterior distributions of the parameters. Data augmentation started at a set of data-driven initiating parameter values. For example, trimmed means and variances across samples were used as the initial values for the parameters in the normal distribution of the mixture. Further details of the Bayesian hierarchical mixture model used here can be found in Parmigiani et al. [8]. Matrices of  were obtained for each of the five data sets (Additional files 1, 2, 3, 4).

### Leave-one-out cross validation and risk index computation

For the combined sample pool of the breast cancer patients (the meta-cohort), we defined outcome groups as recurred during followup and remained relapse-free for at least 3 years. In particular, Let *T*_*i *_be the event time for subject *i*, *C*_*i *_be the censoring time for subject *i*, and *δ*_*i *_= 1{*T*_*i *_<*C*_*i*_} be the censoring indicator. Define a new outcome variable,



where t* can be specified with clinical knowledge. We chose t* = 3 years in this study. We then consider constructing classifiers using *y*; note that *y *= 1 corresponds to the poor outcome group and *y *= 0 to the good outcome group. The sample sizes for each study are shown in Table 1.

Logistic regression was used to build a classifier for prognosis. For each gene *j*, we fit the following univariate logistic regression model using data from all studies:



where x* is the rescaled value that allows data integration across multiple studies. The esti-mated values of *β*_*j*_, , are then used to form a risk score using a variation of the compound covariate predictor method [28,29]; for a given set of covariate values *x*_1_, ..., *x*_*D*_, the risk index is given as .

If we want to assess the performance of the classifier, we must deal with the issue of training and testing the model using the same data. An "honest" estimate of the prediction error rate is obtained using leave-one-out cross-validation. Define a risk index , where , and  is the effect estimate for gene *j *in the combined meta-cohort without the *i*^*th *^sample. The risk index for sample *i *is a weighted linear combination of the expression profiles of the top *D *genes, where the ranking of the genes is based on their corresponding significance in the univariate logistic model fit. Classification of sample *i *to the risk groups is then based on the *i*^*th *^leave-one-out risk index, i.e.,  = *I*{*RI*_*i *_>*c*} with *c *being the empirical quantiles (40^*th *^- 70^*th*^) of the *RI's*. The number of genes *D *in a classifier is treated as a parameter and optimized to minimize the prediction error rates.

The list of the top cumulative genes in the meta-signature was obtained by ranking all 2,555 genes by the number of times in the leave-one-out cross-validation steps that each one had a P-value from the univariate logistic regression less than 0.001.

### Heat map display

We used the treeview software [30] to generate a heat map representation of the *poe *pro-files of the meta-signature. Yellow represents high probability of over-expression and blue represents high probability of under-expression. For heat maps of raw data matrices, we preprocessed the data by mean centering and then dividing by the standard deviation for each row. The means and the standard deviations used in the normalization were the relapse-free (RF) sample means and variances for each study data. The values for the recurrence (R) samples after standardizing then represented the number of standard deviations over or under the mean RF sample expression.

## Authors' contributions

RS, DG, and AC designed the study. RS carried out the statistical analysis and prepared the manuscript. DG and AC supervised the analysis and participated in the manuscript preparation. All authors read and approved the final manuscript.

## Supplementary Material

Additional File 5 Plots of misclassification rates. The PDF file lists plots of misclassification error rates for classifiers identified in each individual study cohort and the meta-cohort. Click here for file

Additional File 6 Plots of Kaplan-Meier curves. The PDF file lists Kaplan-Meier plots for study-wise cross-validation of the individually identified signatures. A gene signature was trained in one study cohort and used to validate in each of the other study cohorts as testing sets. Click here for file

Additional File 7 Meta-signature gene list. The excel file contains a list of Unigene ID, gene symbol, and full name of the 90 genes in the meta-signature. Click here for file

Additional File 1 POE imputation of the Sorlie data. The excel file contains a table of imputed signed probability matrix transformed from the Sorlie *et al. *study data (2,555 times 58 in dimension). Click here for file

Additional File 2 POE imputation of the van't Veer data. The excel file contains a table of imputed signed probability matrix transformed from the van't Veer *et al. *study data (2,555 times 78 in dimension). Click here for file

Additional File 3 POE imputation of the Sotiriou data. The excel file contains a table of imputed signed probability matrix transformed from the Sotiriou *et al. *study data (2,555 times 98 in dimension). Click here for file

Additional File 4 POE imputation of the Huang data. The excel file contains a table of imputed signed probability matrix transformed from the Huang *et al. *study data (2,555 times 71 in dimension). Click here for file
