# Supplementary material for: Prognostic meta-signature of breast cancer developed by two-stage mixture modeling of microarray data
Source: BMC Genomics. 2004 Dec 14;5:94. doi: 10.1186/1471-2164-5-94 (PMC544889; doi:10.1186/1471-2164-5-94)
Supplement: Additional File 5 — Plots of misclassification rates. The PDF file lists plots of misclassification error rates for classifiers identified in each individual study cohort and the meta-cohort. [file 1471-2164-5-94-S5.PDF]

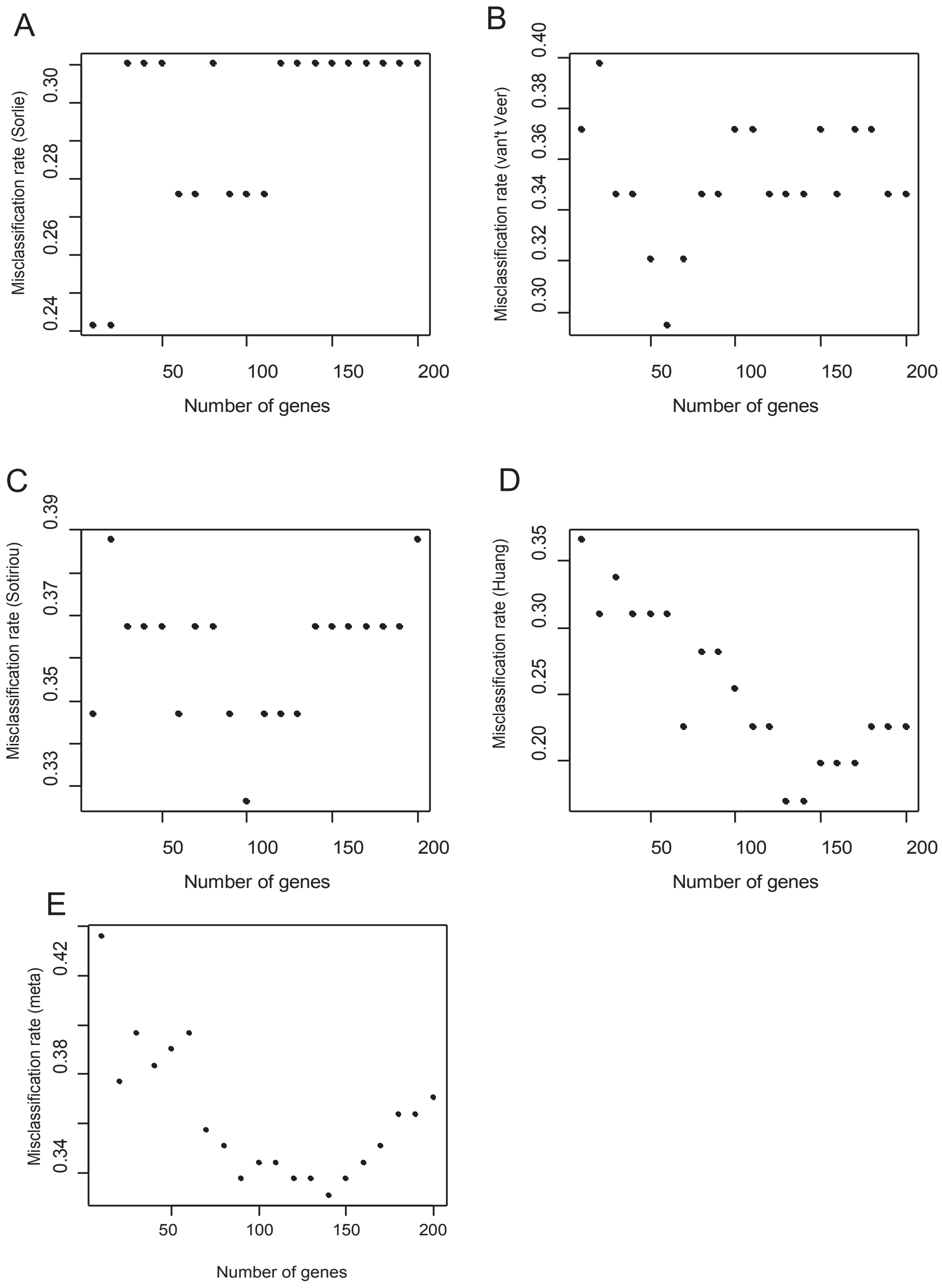

Supplementary Figure 1. Misclassification rates using study-specific gene signatures and the meta-signature of size 10 to 200. A. Sorlie et al. study signature (using 40th percentile of the risk indices as cutoff), B. van't Veer et al. study signature (using 60th percentile RI cutoff), C. Sotiriou et al. study signature (using 50th percentile RI cutoff), D. Huang et al. study signature (using 50th percentile RI cutoff), and E. the meta cohort signatures (using 50th percentile RI cutoff).
