# Supplementary material for: Prognostic meta-signature of breast cancer developed by two-stage mixture modeling of microarray data
Source: BMC Genomics. 2004 Dec 14;5:94. doi: 10.1186/1471-2164-5-94 (PMC544889; doi:10.1186/1471-2164-5-94)
Supplement: Additional File 6 — Plots of Kaplan-Meier curves. The PDF file lists Kaplan-Meier plots for study-wise cross-validation of the individually identified signatures. A gene signature was trained in one study cohort and used to validate in each of the other study cohorts as testing sets. [file 1471-2164-5-94-S6.PDF]

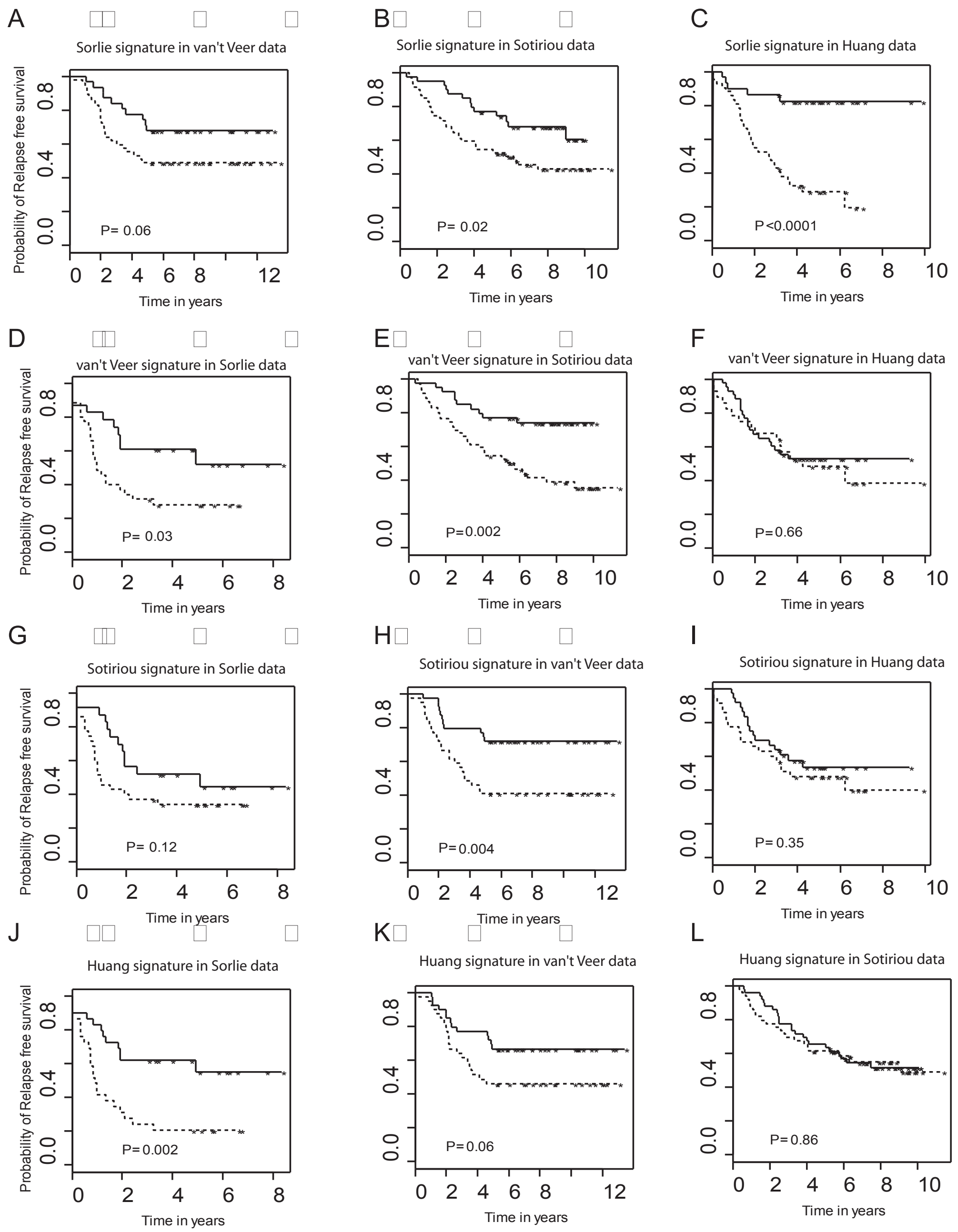

Supplementary Figure 2. Study-wise cross-validation of the individually identified signatures. A gene signature was trained in one study cohort and used to validate in each of the other study cohorts as testing sets.

-The Sorlie signature (10-gene, 40th percentile RI cutoff) was used to predict survival in the van't Veer data (A), the Sotiriou data (B), and the Huang data (C).

-The van't Veer signature (60-gene, 60th percentile RI cutoff) was used to predict survival in the Sorlie data (D), the Sotiriou data (E), and the Huang data (F).

-The Sotiriou signature (100-gene, 50th percentile RI cutoff) was used to predict survival in the Sorlie data (G), the van't Veer data (H), and the Huang data (I).

-The Huang signature (130-gene, 50th percentile RI cutoff) was used to predict survival in the Sorlie data (J), the van't Veer data (K), and the Sotiriou data (L).
